# Reproducibility of Age at Menarche Gathered From Mail Questionnaires: A Report From the Life Span Study

**DOI:** 10.2188/jea.JE20250027

**Published:** 2025-11-05

**Authors:** Yuko Kadowaki, Alina V. Brenner, Michiko Yamada, Hiromi Sugiyama, Mai Utada, Munechika Misumi, Ritsu Sakata

**Affiliations:** 1 Department of Epidemiology, Radiation Effects Research Foundation, Hiroshima, Japan; 2 Department of Clinical Studies, Radiation Effects Research Foundation, Hiroshima and Nagasaki, Japan; 3 Department of Statistics, Radiation Effects Research Foundation, Hiroshima, Japan

**Keywords:** age at menarche, reproducibility, self-report, proxy report, Life Span Study

## Abstract

**Background:**

Menarche timing may affect female health. While previous studies evaluated self-reported age at menarche reproducibility, they did not assess types of respondents. This study compared the reproducibility of age at menarche among self-responders and proxy respondents and assessed proxy-respondent reproducibility by relationship and survey age.

**Methods:**

Data on age at menarche reported in both the 1969 and 1978 mail questionnaires among 9,043 females from the Life Span Study cohort of atomic bomb survivors were analyzed. The reproducibility of menarcheal age was assessed by the type of respondents, by proxy’s relationship to participant, and by age at the 1969 survey using Bland-Altman’s method and the intraclass correlation coefficient (ICC).

**Results:**

Reproducibility was moderate (95% limits of agreement, −2.3 to 2.4 years; ICC 0.72; 95% confidence interval, 0.71**–**0.73). Both self-respondents (*N* = 6,664) and the total study population (*N* = 9,043) maintained moderate reproducibility even at older ages. Groups with proxy reports showed lower reproducibility than self-respondents, with spouse proxy reports showing highest reproducibility and parent proxy reports showing lowest reproducibility among proxy reports, although the comparisons are based on different survey ages in 1969.

**Conclusion:**

This is the first study to evaluate menarcheal-age reproducibility between self- and proxy-reports using appropriate measures. Mail questionnaires at 9-year interval showed moderate reproducibility across all ages, including elderly self-respondents. Reproducibility varied by the respondent–target relationship, with spouse proxies showing highest and parent proxies showing lowest reproducibility among proxy reports. Additional data are required to establish appropriate methods for handling specific proxy responses.

## INTRODUCTION

The reproductive life of females, including the onset of menarche and menopause, has significant health implications. These reproductive events can influence the risks of breast and uterine corpus cancers.^1^^,^^2^ It also affects risks of non-cancer disease incidence and mortality, marking key biological changes.^3^^,^^4^ The timing of menarche typically occurs in adolescence. As a result, recalling the age at menarche in adulthood necessitates reflecting distant memories. Furthermore, when the age at menarche is collected using mail questionnaire survey, reproducibility may differ depending on whether the respondent is the participant herself or a proxy, and in the case of proxies, his/her relationship to the participant. To date, only a few studies have investigated the difference in reproducibility of information about age at menarche by the type of respondent.

A previous study examining the reproducibility of the self-reported age at menarche found that the reproducibility of age at menarche in older females (55 to 73 years old at the time of the survey) was comparable to that in younger females.^5^ Although one study included participants aged up to 89 years, the interval between surveys was relatively short at 2.5 years,^6^ limiting the assessment of long-term reproducibility in elderly populations. Regarding proxy reports, while some studies have evaluated reproducibility for specific relationships, such as spouses or twins, comprehensive assessment across different proxy types has been limited.^7^^,^^8^

Reproducibility can be assessed in several ways. Reliability of reporting is often evaluated using the intraclass correlation coefficient (ICC), while agreement is evaluated by the difference between two measurements.^9^ Previous studies have sometimes used Pearson’s correlation coefficient to assess reliability of the reported age at menarche.^5^^,^^10^ This approach, however, fundamentally is a way to measure the correlation, which does not make it the most accurate indicator of reliability.^11^

The Life Span Study (LSS) of atomic bomb survivors provides an opportunity to evaluate the reproducibility of the age at menarche reported in the 1969 and 1978 mail questionnaire surveys. Both surveys covered a wide age range of LSS participants (23 to 88 years in 1969, and 32 to 97 years in 1978) and allowed the information to be reported by proxy respondents. The 1969 survey also collected information on the relationship of proxy to the participant.

The objective of this study was to evaluate the reproducibility of age at menarche gathered from 1969 and 1978 mail questionnaires in the LSS cohort. Additionally, we assessed whether the reproducibility of the age at menarche varied by the age of the participants at the time of the 1969 survey, by the type of respondent, and by the relationship of proxy to participant.

## METHODS

### Study subjects

The LSS cohort has 120,321 members, of which 70,146 are females. Of these, 59,078 females either did not respond or responded to one of the two mail questionnaires conducted in 1969 and 1978; 11,068 females responded to both questionnaires and could potentially be used for reproducibility analysis (eFigure 1). The final subjects for analysis consisted of 9,043 females, after excluding participants who did not state their age at menarche in either survey (*N* = 1,925), and those who reported having their first menstrual period at age 20 or older in either survey (*N* = 100).

### Mail questionnaires

The 1969 survey was mailed to all females in the LSS known to be alive at that time, excluding those who were members of the biennial health-examination program. The 1978 survey was mailed to all females in the LSS cohort, excluding the non-exposed (individuals who were not in Hiroshima and Nagasaki cities at the time of the bombings). The reference dates for these surveys were designated as January 1, 1969, and September 1, 1978, respectively. The relevant question in the 1969 survey was “Menstruation: Age at menarche (in whole years)”, while in the 1978 survey, it was “About menstruation: When did your menstruation begin? At age.” In both instances, the age at menarche was documented in whole numbers. A question regarding who the type of respondent was (ie, the LSS participant or a proxy) and, in case of a proxy, a further question about the proxy’s relationship to the participant were included in both questionnaires. For the 1978 questionnaire, however, the database did not include information about the relationship between proxies and participants.

### Types of respondents

In the analysis, respondents were categorized into four groups: (1) self-respondents to both surveys, (2) self-respondent in 1969 and proxy-respondent in 1978, (3) proxy-respondent in 1969 and self-respondent in 1978, and (4) other combinations of respondents, which included proxy-respondents to both surveys and unspecified respondents to either survey.

### Relationships of proxies to participants

The proxy respondents to 1969 questionnaire were stored in the RERF database as follows: parent, spouse, child, sibling, grandchild, and other. Due to small number for sibling, grandchild, and other, these three categories were combined into a single category labeled “other” for analysis purposes.

### Statistical analysis

Reproducibility can be assessed in several ways. To evaluate agreement in the reported ages at menarche, the 95% limits of agreement were calculated using Bland-Altman’s method.^12^ Reliability was determined using the ICC, which was estimated using a two-way mixed-effects model corresponding to ICC (3,1), treating survey as a fixed effect and respondent as a random effect.^13^ The consistency type was selected for test-retest reliability assessment. Both the 95% limits of agreement and ICC were calculated from separate mixed-effects models fit within each stratum defined by (1) type of respondents including proxy respondents in 1969, and (2) participant’s age at the 1969 survey (23**–**30, 31**–**40, 41**–**50, 51**–**60, and 61**–**88 years). For the ICC, a 95% lower confidence limit at or above 0.90 was interpreted as “Excellent” reliability, while values from below 0.90 to 0.75 indicate “Good” reliability, from below 0.75 to 0.5 denote “Moderate” reliability and values below 0.50 are categorized as “Poor” reliability.^11^ Prior to applying Bland-Altman’s method and ICC, the normality of the distribution of differences between reported ages at menarche was evaluated visually and separately for types of respondents, relationships of proxies to participants, and age groups using Q-Q plots and histograms. The normality of the distribution of differences between reported ages at menarche was not perfectly normal. However, it did not differ markedly from normality, so Bland-Altman’s method and ICC were applied without variable transformation (data not shown).

Additionally, to perform visual inspection of patterns in the differences of reported age at menarche between the two surveys, stratified Bland-Altman plots were created by the type of respondents for all reports and by relationship of proxy to participant for proxy respondents in 1969.

Statistical analyses were performed using RStudio version 2023.09.1 and R version 4.3.1 (R Foundation for Statistical Computing, Vienna, Austria). The “irr” package was used for the ICC analysis, including calculating its 95% confidence intervals (CIs).^13^^,^^14^

### Ethics approval

This study was approved by the Institutional Review Board of the Radiation Effects Research Foundation (RERF) (Research Protocol 1-75). Informed consent could not be obtained due to the inability to contact the participants. Instead, study information was provided to participants through the RERF webpage.

## RESULTS

Table 1 summarizes the number and percentage of subjects by age in the 1969 survey and the types of respondents. The 1969 age range was 23**–**88 years. The number of study subjects gradually increased at ages 23**–**30 years, reaching its peak at ages 41**–**50 years, and then declined. Among those who were proxy-respondent in 1969 and self-respondent in 1978, parents were the primary proxy respondents for younger participants (70.2% in the 23**–**30 years age group), spouses were more common for middle-aged participants, and children were the main proxy respondents for older participants (52.5% in the 61**–**88 years age group). Additionally, the percentage of self-respondents to both surveys decreased with increasing age after the 41**–**50 years age groups.

**Table 1.** Distribution of study subjects who reported age at menarche in both surveys in 1969 and 1978 by type of respondents and age at the 1969 survey

Respondent	Age at survey in the 1969, years^a^					Total

	23–30	31–40	41–50	51–60	61–88	
Self-respondents to both surveys	1,387 (79.3)	1,794 (83.2)	1,845 (79.5)	1,097 (68.0)	541 (45.0)	6,664 (73.7)
Self-respondent in 1969 and proxy-respondent in 1978	15 (0.9)	20 (0.9)	66 (2.8)	124 (7.7)	201 (16.7)	426 (4.7)
Proxy-respondent in 1969 and self-respondent in 1978	124 (7.1)	45 (2.1)	40 (1.7)	46 (2.9)	59 (4.9)	314 (3.5)

Parent	87 (70.2)	7 (15.6)	3 (7.5)	1 (2.2)	0 (0.0)	98 (31.2)
Spouse	12 (9.7)	24 (53.3)	20 (50.0)	17 (37.0)	13 (22.0)	86 (27.4)
Child	1 (0.8)	1 (2.2)	11 (27.5)	21 (45.7)	31 (52.5)	65 (20.7)
Other	24 (19.4)	13 (28.9)	6 (15.0)	7 (15.2)	15 (25.4)	65 (20.7)

Other combinations of respondents	224 (12.8)	297 (13.8)	371 (16.0)	347 (21.5)	400 (33.3)	1,639 (18.1)

**Total**	1,750 (100.0)	2,156 (100.0)	2,322 (100.0)	1,614 (100.0)	1,201 (100.0)	9,043 (100.0)

^a^*N* and % in the parenthesis.

Overall, the mean age at the 1969 survey was 44.1 years, and the mean age at menarche was 14.8 years (Table 2). The mean difference in age at menarche (1978 minus 1969) was 0.03 (standard deviation [SD], 1.2) years, the 95% limits of agreement for reported age at menarche ranged from −2.3 to 2.4 years, and the ICC showed moderate reliability (ICC 0.72; 95% lower confidence limit, 0.71) (Table 2, Table 3, and Table 4). Of all participants, 44.4% reported the same age at menarche in both surveys, while 4.8% showed a difference of more than 2 years between the surveys (data not shown). The mean difference and SD in age at menarche and the measures of reproducibility—the 95% limits of agreement for reported age at menarche and ICC—showed similar values across all types of respondent except for the proxy-respondent in 1969 and self-respondent in 1978 (Table 2, Table 3, and Table 4).

**Table 2.** Comparison of characteristics of participants by combination of type of respondents

Parameters	Self-respondents to both surveys	Self-respondent in 1969 and proxy-respondent in 1978	Proxy-respondent in 1969 and self-respondent in 1978	Other combinations of respondents	Overall
	(*N* = 6,664)	(*N* = 426)	(*N* = 314)	(*N* = 1,639)	(*N* = 9,043)
**Age at the 1969 survey, years** ^a^	42.2 (11.9)	58.0 (11.7)	42.3 (16.1)	48.6 (14.1)	44.1 (13.1)
**Age at menarche in 1969, years** ^a^	14.6 (1.6)	15.3 (1.6)	15.0 (1.6)	15.1 (1.7)	14.8 (1.6)
**Age at menarche in 1978, years** ^a^	14.7 (1.6)	15.4 (1.6)	14.7 (1.7)	15.1 (1.7)	14.8 (1.6)
**Difference in age at menarche between 1969 and 1978 surveys, years** ^a^	0.04 (1.2)	0.09 (1.3)	−0.34 (1.6)	0.02 (1.3)	0.03 (1.2)

^a^Mean and standard deviation in the parenthesis.

**Table 3.** 95% limits of agreement for reported age at menarche (year) between both surveys in 1969 and 1978 by type of respondents and age at the 1969 survey

Type of respondent	Age at the 1969 survey, years					Total

	23–30	31–40	41–50	51–60	61–88	
Self-respondents to both surveys	−2.2 to 1.8	−2.3 to 2.3	−2.1 to 2.5	−2.1 to 2.2	−2.2 to 2.7	−2.2 to 2.3
Self-respondent in 1969 and proxy-respondent in 1978	−2.1 to 3.8	−2.2 to 2.1	−1.9 to 2.2	−2.0 to 2.6	−2.9 to 2.6	−2.4 to 2.6
Proxy-respondent in 1969 and self-respondent in 1978	−3.9 to 2.0	−2.4 to 2.2	−2.9 to 2.5	−3.1 to 3.9	−2.8 to 3.0	−3.4 to 2.8

Parent	−4.1 to 2.0	−2.8 to 2.5	−4.9 to 6.9	NA to NA	NA to NA	−4.1 to 2.3
Spouse	−1.8 to 0.8	−2.5 to 2.4	−2.7 to 2.4	−3.2 to 3.1	−2.3 to 2.8	−2.6 to 2.4
Child	NA to NA	NA to NA	−2.1 to 1.2	−1.9 to 3.8	−3.1 to 3.4	−2.8 to 3.4
Other	−4.0 to 2.3	−2.2 to 1.9	−3.7 to 2.7	−5.3 to 5.9	−2.6 to 2.3	−3.6 to 2.8

Other combinations of respondents	−2.6 to 2.2	−2.6 to 2.6	−2.2 to 2.6	−2.2 to 2.5	−2.8 to 2.7	−2.5 to 2.5

**Total**	−2.4 to 1.9	−2.4 to 2.3	−2.1 to 2.5	−2.2 to 2.4	−2.6 to 2.7	−2.3 to 2.4

NA, not available due to no participants or only one participant.

**Table 4.** Intraclass correlation coefficient (ICC) among reported age at menarche in both surveys in 1969 and 1978 by type of respondents and age at the 1969 survey

Type of respondent	Age at the 1969 survey, years^a^					Total

	23–30	31–40	41–50	51–60	61–88	
Self-respondents to both surveys	0.65 (0.62**–**0.68)	0.73 (0.71–0.75)	0.73 (0.71–0.75)	0.73 (0.70–0.75)	0.65 (0.60–0.69)	0.74 (0.73–0.75)
Self-respondent in 1969 and proxy-respondent in 1978	0.26 (−0.27 to 0.67)	0.72 (0.42–0.88)	0.83 (0.73–0.89)	0.71 (0.61–0.79)	0.57 (0.47–0.66)	0.67 (0.61–0.72)
Proxy-respondent in 1969 and self-respondent in 1978	0.38 (0.21**–**0.52)	0.75 (0.59–0.86)	0.58 (0.33–0.76)	0.35 (0.07–0.58)	0.59 (0.40–0.74)	0.55 (0.47–0.62)

Parent	0.32 (0.11–0.49)	0.56 (−0.24 to 0.91)	0.23 (−0.92 to 0.97)	NA	NA	0.32 (0.13–0.49)
Spouse	0.88 (0.64–0.96)	0.79 (0.57–0.90)	0.67 (0.33–0.86)	0.50 (0.04–0.78)	0.57 (0.06–0.85)	0.70 (0.57–0.79)
Child	NA	NA	0.81 (0.45–0.95)	0.44 (0.02–0.73)	0.57 (0.27–0.76)	0.56 (0.37–0.71)
Other	0.34 (−0.06 to 0.65)	0.66 (0.19–0.88)	0.26 (−0.62 to 0.85)	0.11 (−0.65 to 0.76)	0.62 (0.17–0.85)	0.51 (0.31–0.67)

Other combinations of respondents	0.58 (0.49–0.66)	0.70 (0.63–0.75)	0.72 (0.66–0.76)	0.72 (0.66–0.76)	0.59 (0.52–0.65)	0.70 (0.68–0.73)

**Total**	0.60 (0.57–0.63)	0.73 (0.71–0.75)	0.73 (0.71–0.75)	0.71 (0.69–0.74)	0.61 (0.57–0.64)	0.72 (0.71–0.73)

NA, not available due to no participants or only one participant.

^a^ICC and 95% confidence interval in the parenthesis.

In the proxy-respondent in 1969 and self-respondent in 1978, by relationship of proxy to participant, the mean age at the 1969 survey increased in the following order: parent, spouse, and child, with the mean age at menarche showing a similar pattern (eTable 1). Parent proxies in 1969 reported later ages at menarche compared to self-reports in 1978, showing the largest difference of age at menarche between the two surveys. When comparing the results of stratified analyses, the 95% limits of agreement for reported age at menarche were wider for parent proxy reports than for spouse or child proxy reports, and the ICC showed poor reliability for parent proxies while maintaining moderate reliability for spouse or child proxies (Table 3 and Table 4).

**Figure 1.**
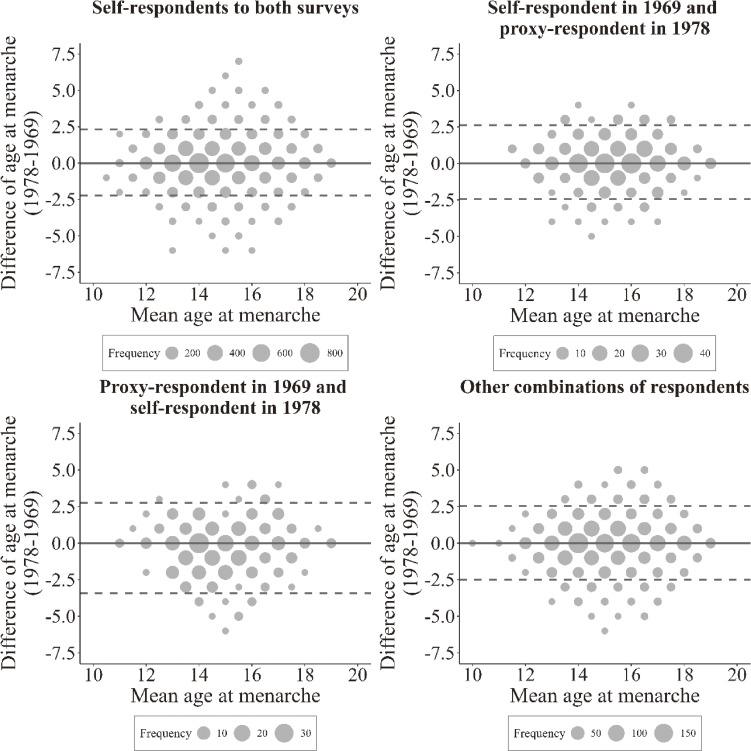
The Bland-Altman plot of difference and mean of age at menarche between 1969 and 1978 surveys by type of respondents. The y-axis shows the difference in age at menarche between the two surveys (1978 minus 1969), while the x-axis represents the mean of age at menarche between the two surveys. The solid line represents zero difference in age at menarche, and the long-dashed lines show the 95% upper and lower limits of agreement. The difference in age at menarche shows age at menarche in 1978 minus age at menarche in 1969.

The Bland-Altman plots for reported age at menarche in proxy-respondent in 1969 and self-respondent in 1978 indicated that proxy-reported ages tended to be later than self-reported ages (Figure 1), which is a distribution with more negative differences. Other types of respondents showed similar distribution with almost equal positive and negative differences.

In the Bland-Altman plots by relationship of proxy to participant for the proxy-respondent in 1969 and self-respondent in 1978 (eFigure 2), parent proxy reports showed more negative differences when the mean age at menarche was below 15 years. In contrast, child proxy reports showed more positive differences when the mean age at menarche was above 15 years.

The effects of age at survey were examined in self-respondents to both surveys, as there were sufficient numbers of participants in each age category. The 95% limits of agreement remained relatively stable, ranging from −2.2 to 2.3 years overall and from −2.2 to 2.7 years even in the oldest age group (61**–**88 years) (Table 3). The reliability also remained moderate across all age groups, with the ICC being 0.74 (95% lower confidence limit, 0.73) overall and 0.65 (95% lower confidence limit, 0.60) in the 61**–**88 years age group (Table 4). Similar stability in reproducibility across age groups was observed in the total study population (Table 3 and Table 4). Additionally, in the total study population, females in younger age groups reported an earlier age at menarche compared to those in older age groups (by 1.7 years in 1969 and 1.9 years in 1978), indicating that females in younger birth cohorts experienced menarche at earlier ages (data not shown).

## DISCUSSION

Analysis of data from nearly 9,000 LSS females who provided the age at menarche information in two mail questionnaire surveys conducted at a 9-year interval showed 95% limits of agreement ranging from −2.3 to 2.4 years with moderate reliability (ICC, 0.74). Table 5 compares the reproducibility of self-reported age at menarche between two prior studies, each including over 1,000 participants, and self-respondents to both surveys in the present study. Despite this extended period (9 years) in this analysis, the reliability remained moderate and was comparable to the previous studies with shorter intervals. Possible interpretations are that the LSS participants who are atomic bomb survivors may maintain a reliable recall of menarche over longer time and that the recall of age at menarche may be relatively robust across different populations and experiences. Because it is difficult to distinguish at this time, caution is warranted in generalizing these findings to populations with markedly different backgrounds or survey conditions.

**Table 5.** Comparison of the results of reproducibility studies

Study	This study	Lundblad et al (2017)	Schmidt-Pokrzywniak et al (2016)
**Country**	Japan	Norway	Germany
**Year of the first survey**	1969	1986**–**1987	2006**–**2010
**Number of respondents to the question of self-reported age at menarche ** **for both surveys**	6,664	6,731	1,670
**Age range at survey** **(Ordinal number of survey)**	23**–**88 (1st)	25**–**73 (2nd)	18**–**89 (1st)
**Interval of surveys, years**	9	7	2.5^a^
**Method of survey**	Mail questionnaire	Self-administered questionnaire at physical examination	Mail questionnaire
**Additional query for missing or suspicious responses with the participant**	No	Not described	Yes
**Pearson’s correlation coefficient (overall)**	Not described	0.84	Not described
**Degree of reliability by ICC (overall)**	Moderate	Not described	Moderate
**95% limits of agreement (overall)**	−2.2 to 2.3	−1.52 to 1.54	>−2 to <2

ICC, intraclass correlation coefficients.

^a^Median.

Regarding the agreement in the proxy reports, the Israeli study that assessed the agreement of the age at menarche surveying middle-aged females and their husbands by telephone interview reported that use of spouse as a proxy respondent for reporting the age at menarche should be carefully considered.^7^ The present finding that spouse proxies reports showed relatively high agreement contrasts with the findings from the Israeli study.^7^ These differences in conclusions might be attributable to differences in study populations (Japanese atomic bomb survivors versus middle-aged and older females visiting an Israeli hospital and their husbands), methods of collecting age at menarche information (mail questionnaire survey versus telephone interview), and sample sizes (86 vs 14 couples).

Among those who were proxy-respondent in 1969 and self-respondent in 1978, child proxy reports showed slightly earlier mean age at menarche than self-reports in 1978, whereas parent proxy reports showed later mean age at menarche than self-reports in 1978. The proxy respondent’s own age at menarche might be reflected in the above-mentioned differences, because the proxy respondent in older generation experienced later age at menarche. However, interpretation of these proxy reports is limited by data collection constraints in the present study. The RERF database used in this study stored parent proxies without distinguishing between mothers and fathers, and child proxies without distinguishing between sons and daughters. Mothers may report age at menarche more accurately than fathers, and daughters may provide more accurate reports than sons. Similar considerations apply to siblings and other relatives. The differences in reported age at menarche observed between proxy respondents may be influenced by various factors, including generational differences in recall, family communication patterns, and potentially the proxy respondents’ own experiences because age at menarche decreased in younger birth years. However, without more detailed data on proxy respondent characteristics, it is difficult to draw firm conclusions about the reasons for these differences. Furthermore, it should be noted that older participants were more likely to require proxy respondents, and the relationship of proxies to participants changed with age. The differences in reproducibility observed between types of proxy relationships may be partly influenced by the age factor of the participants.

One important limitation of the study is that the validity of the reported age at menarche could not be evaluated due to the absence of information about the actual age at menarche (eg, from prospectively collected diaries or medical records). Additionally, a clear definition of menarche (eg, the age at first menstrual period vs the age at which menstrual cycles become regular) was not provided in the questionnaires, which may have led to variability in interpretations among respondents. However, since similar question formats were used in both surveys, the impact of this lack of clarity on the reproducibility assessment is likely limited.

An important strength of the study is that it is the first to evaluate differences in reproducibility of age at menarche between self-reports and proxy-reports using agreement and reliability. These findings are meaningful for large-scale epidemiologic surveys using mail questionnaires. The investigation of reproducibility across proxy relationships may help to identify reliable proxies and to improve response selection for analysis.

In conclusion, age at menarche from the mail questionnaire surveys conducted at 9-year intervals showed moderate reproducibility across all age groups, including the elderly, in both self-respondents to both surveys and the total study population. Significantly, older participants were more likely to rely on proxies. The reproducibility differed according to the relationship between proxy respondents and the participants. However, detailed distinctions could not be made. Additional data are required to establish appropriate methods for handling specific proxy responses.

## SUPPLEMENTARY MATERIAL

eTable 1. Comparison of characteristics of participants by relationship of proxy to participant in the proxy-respondent in 1969 and self-respondent in 1978 group

eFigure 1. Selection process of study subjects

eFigure 2. The Bland-Altman plot of difference and mean of age at menarche between 1969 and 1978 surveys by relationship of proxy to participant for the proxy-respondent in 1969 and self-respondent in 1978. The difference in age at menarche between the two surveys (1978 minus 1969) was plotted on the y-axis and the mean of age at menarche of the two surveys was plotted on the x-axis. The solid line represents zero difference in age at menarche, and the long dashed lines show the 95% upper and lower limits of agreement.
