# Supplementary material for: Reproducibility of Age at Menarche Gathered From Mail Questionnaires: A Report From the Life Span Study
Source: J Epidemiol. 2025 Nov 5;35(11):490–5. doi: 10.2188/jea.JE20250027 (PMC12527403; doi:10.2188/jea.JE20250027)

eTable 1. Comparison of characteristics of participants by relationship of proxy to participant in the proxy-respondent in 1969 and self-respondent in 1978 group

Parameters	Relationship of proxy to participant			
	**Parent (N=98)**	**Spouse (N=86)**	**Child (N=65)**	**Other (N=65)**
**Age at the 1969 survey, years [a]**	27.7 (5.3)	45.4 (11.9)	59.3 (10.3)	43.1 (17.5)
**Age at menarche in 1969, years [a]**	14.6 (1.5)	15.1 (1.6)	15.4 (1.7)	15.2 (1.6)
**Age at menarche in 1978, years [a]**	13.7 (1.2)	15.0 (1.7)	15.6 (1.7)	14.8 (1.6)
**Difference in age between 1969 and 1978 surveys, years [a]**	-0.91 (1.6)	-0.10 (1.3)	0.28 (1.6)	-0.40 (1.6)

[a] Mean and standard deviation in the parenthesis

eFigure 1. Selection process of study subjects

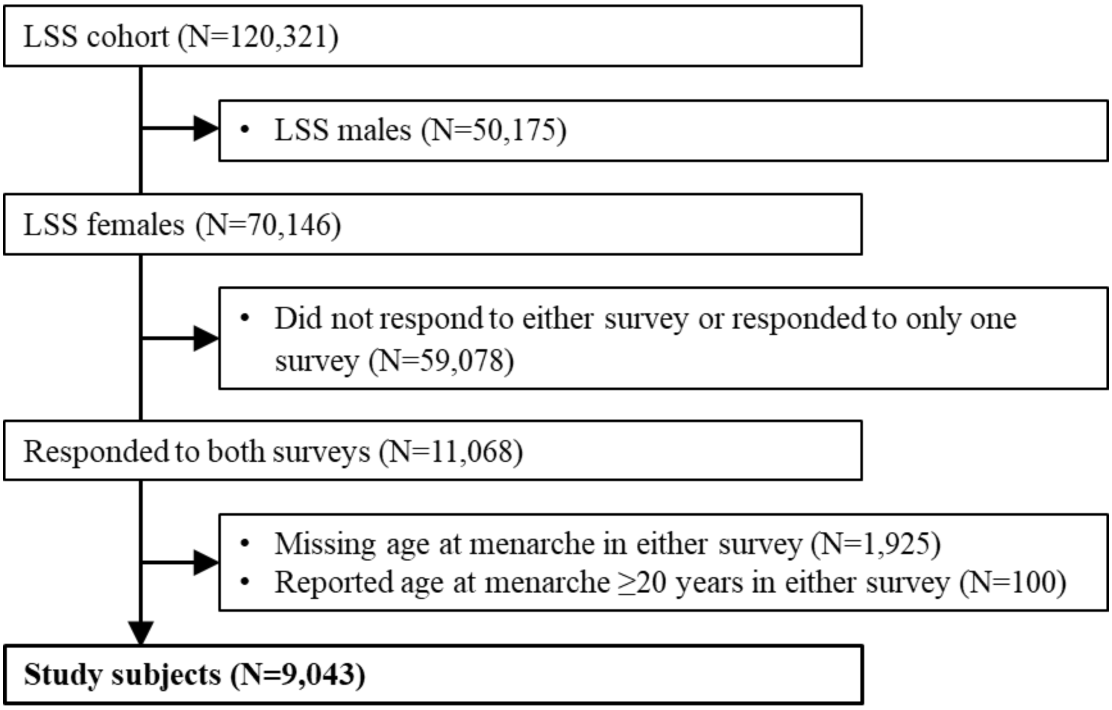

eFigure 2. The Bland-Altman plot of difference and mean of age at menarche between 1969 and 1978 surveys by relationship of proxy to participant for the proxy-respondent in 1969 and self-respondent in 1978. The difference in age at menarche between the two surveys (1978 minus 1969) was plotted on the y-axis and the mean of age at menarche of the two surveys was plotted on the x-axis. The solid line represents zero difference in age at menarche, and the long dashed lines show the 95% upper and lower limits of agreement.

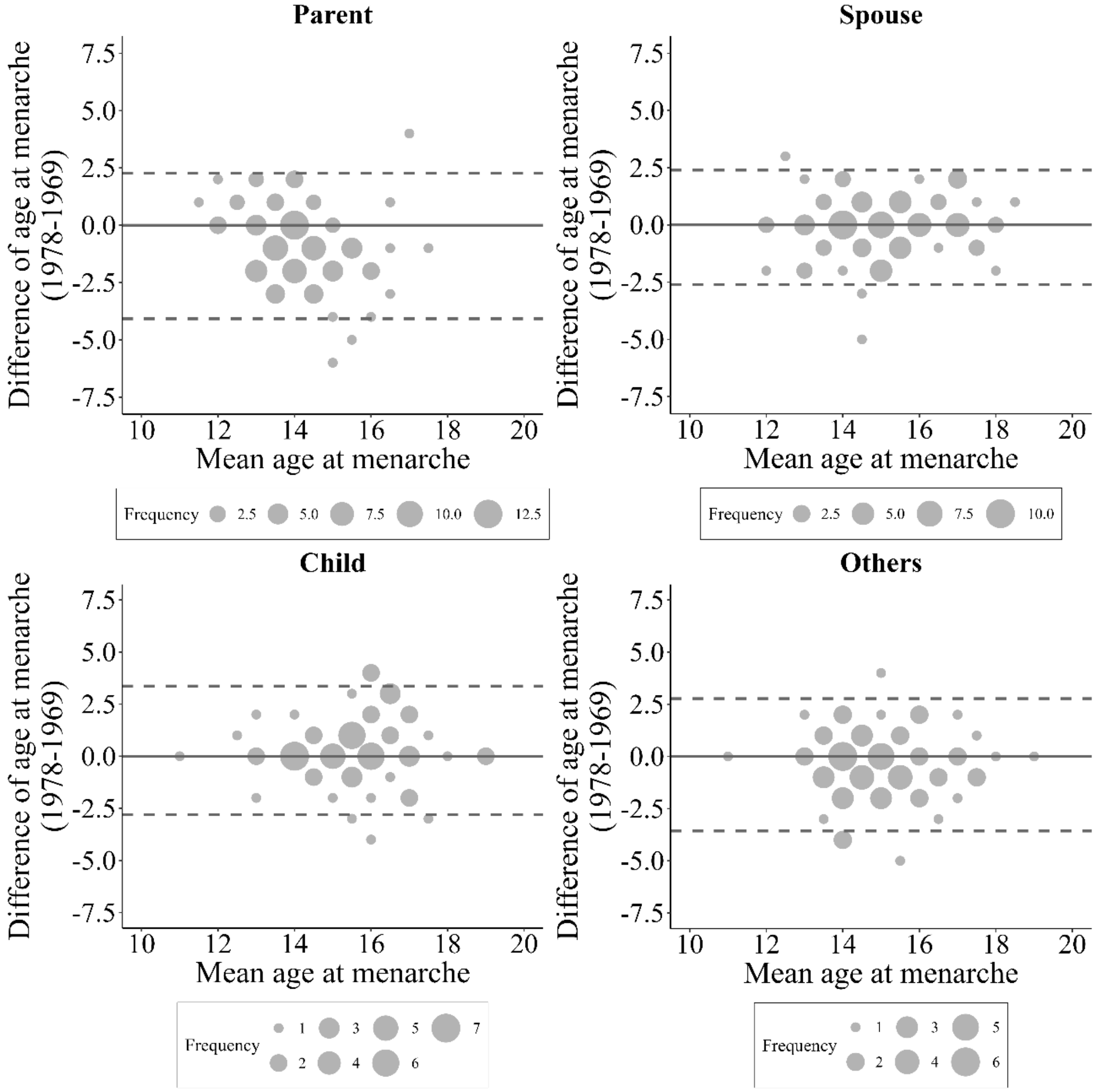

Supplement: Supplementary file 1 [file je-35-490-s001.pdf]
